# Absorbed dose and dose rate using the Varian OBI 1.3 and 1.4 CBCT system

**DOI:** 10.1120/jacmp.v11i1.3085

**Published:** 2010-01-28

**Authors:** Åsa Palm, Elisabeth Nilsson, Lars Herrnsdorf

**Affiliations:** ^1^ Sahlgrenska University Hospital Deptartment of Medical Physics and Biomedical Engineering Göteborg Sweden; ^2^ RTI Electronics AB Mölndal Sweden

**Keywords:** Cone‐beam CT, absorbed dose, TLD, CT Dose Profiler

## Abstract

According to published data, the absorbed dose used for a CBCT image acquisition with Varian OBI v1.3 can be as high as 100 mGy. In 2008 Varian released a new OBI version (v1.4), which promised to reduce the imaging dose. In this study, absorbed doses used for CBCT image acquisitions with the default irradiation techniques of Varian OBI v1.3 and v1.4 are measured.

TLDs are used to derive dose distributions at three planes inside an anthropomorphic phantom. In addition, point doses and dose profiles inside a ‘stack’ of three CTDI body phantoms are measured using a new solid state detector, the CT Dose Profiler. With the CT Dose Profiler, the individual pulses from the X‐ray tube are also studied. To verify the absorbed dose measured with the CT Dose Profiler, it is compared to TLD. The image quality is evaluated using a Catphan phantom.

For OBI v1.3, doses measured in transverse planes of the Alderson phantom range between 64 mGy and 144 mGy. The average dose is around 100 mGy. For OBI v1.4, doses measured in transverse planes of the Alderson phantom range between 1 mGy and 51 mGy. Mean doses range between 3‐35 mGy depending on CBCT mode. CT Dose Profiler data agree with TLD measurements in a CTDI phantom within the uncertainty of the TLD measurements (estimated SD ±10%). Instantaneous dose rate at the periphery of the phantom can be higher than 20 mGy/s, which is 10 times the dose rate at the center. The spatial resolution in v1.4 is not as high as in v1.3.

In conclusion, measurements show that the imaging doses for default modes in Varian OBI v1.4 CBCT system are significantly lower than in v1.3. The CT Dose Profiler is proven fast and accurate for CBCT applications.

PACS number: 87.53.Bn

## I. INTRODUCTION

Since the introduction of the Varian On‐Board Imager (OBI) system in our clinic in 2007, the request for orthogonal kV and cone‐beam CT (CBCT) images is increasing. However, the absorbed dose used for a CBCT image acquisition with Varian OBI v1.3 can be up to 100 mGy in the head and neck region as well as in the pelvic region, when using the default CBCT modes.^(^ ^1^ ^,^ ^2^ ^,^ ^3^ ^,^ ^4^ ^)^ Based on a 30 fraction treatment, the total physical dose due to CBCT on every fraction can then be 3 Gy. This can often not be accepted, as the treatment plan is optimized based on the tolerance dose of critical structures such as the medulla (currently the imaging dose cannot be included in the treatment planning process). CBCT also contributes dose to normal tissues that are outside the treated region, but within the imaged region.

In 2008 Varian released a new OBI version (v1.4). Dose‐saving improvements include, for example, a change in the convolution filtering which makes better use of the existing data, and the ‘half scan’ option (200° gantry rotation), which allows for a lens saving technique by avoiding imaging at angles $\pm 80^{{}^{\circ}}$ from the front. Varian declares that v1.4 can reduce the imaging dose in the head and neck region to one‐fifth of the dose received with v1.3, for similar image quality, while the dose to the pelvic region can be halved. To our knowledge there is no published dose data on OBI v1.4, other than the values reported by Varian.

In this study, absorbed doses used for CBCT image acquisition with the default irradiation techniques of Varian OBI v1.3 and v1.4 (before and after an upgrade) are measured. TLDs are used to derive dose distributions inside an anthropomorphic phantom. In addition, point doses and dose profiles inside a ‘stack’ of three computed tomography dose index (CTDI) body phantoms are measured using a new solid state detector, the CT Dose Profiler (RTI Electronics AB, Mölndal, Sweden). With the CT Dose Profiler, the individual pulses from the X‐ray tube are also studied. To verify the absorbed dose measured with the CT Dose Profiler, it is compared to TLD.

## II. MATERIALS AND METHODS

### A. Machine and CBCT parameters

All measurements are performed on a Varian Clinac iX Linear accelerator with a kV imaging system (On‐Board Imager). Default CBCT imaging modes are used (see Tables 1 and 2 for details). A beam width of 13.7 cm and 15 cm is used for v1.3 and v1.4, respectively. The field of view is set to 25 cm for the head fan type (also called Full fan) and 45 cm for the body fan type (also called Half fan). Tables 1 and 2 also show ${\text{CTDI}}_{w1}$ values related to each CBCT mode as stated by Varian. The values are based on measurements with a 10 cm long ionization chamber in a standard CTDI phantom. The blades are reduced so that only a narrow field is irradiated, and the narrow beam geometry is then accounted for (personal communication).

**Table 1:** Details of the default CBCT modes with OBI 1.3.

*Mode Name*	*Acquisition Angle [deg]*	*Fan Type [Head/Body]*	*Technique*	*mAs*	*Dose* ${\text{CTDI}}_{w}\;[\text{mGy}]$
Standard Dose	360	Head	125 kV 80 mA 25 ms	1300	90
Standard Dose	360	Body	125 kV 80 mA 25 ms	1300	38
Low Dose	360	Head	125 kV 40 mA 10 ms	260	18
Low Dose	360	Body	125 kV 40 mA 10 ms	260	7.6

Data from the Varian On‐Board Imager (OBI) Reference Guide^(4)^ – except mAs, which is calculated based on 650 projections. Stated measurement uncertainties ±10%.

**Table 2:** Details of the default CBCT modes with OBI 1.4.

*Mode Name*	*Acquisition Angle [deg]*	*Fan Type [Head/Body]*	*Technique*	*mAs*	*Dose* ${\text{CTDI}}_{w}[\text{mGy}]$
Low Dose Head	200	Head	100 kV 10 mA 20 ms	72	2.0
Standard Dose Head	200	Head	100 kV 20 mA 20 ms	145	3.9
High‐quality Head	200	Head	100 kV 80 mA 25 ms	720	19.4
Low Dose Thorax	360	Body	110 kV 20 mA 20 ms	262	4.7
Pelvis Spot Light	200	Head	125 kV 80 mA 25 ms	720	14.4
Pelvis	360	Body	125 kV 80 mA 13 ms	680	17.7

Data from the Varian On‐Board Imager (OBI) Reference Guide.^(4)^ Stated measurement uncertainties ±10%.

It should be mentioned that the operator can create CBCT modes specifically for his/her clinical tasks. The exposure can also be adjusted for an individual patient at the time of CBCT image acquisition. The doses are expected to vary with the chosen settings.

### B. Dose distribution in an anthropomorphic phantom

Absorbed doses for the default CBCT modes are measured in a female Alderson anthropomorphic phantom using TLDs. The dose distribution is measured at three transversal planes: in the head, in the pelvis, and in the thoracic region.

**Figure 1:**
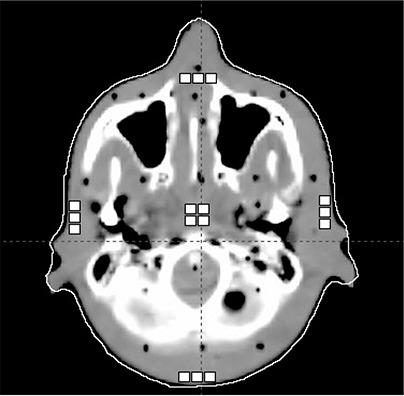
Positions of TLDs (white squares) in the head of the Alderson phantom. Approximate phantom dimension 14×17cm. The central TLDs were positioned at isocenter.

**Figure 2:**
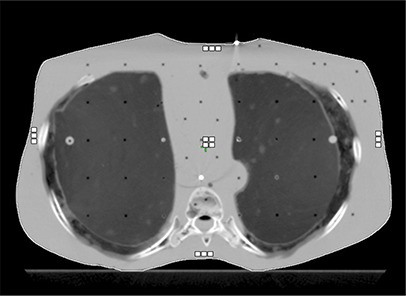
Positions of TLDs (white squares) in the thoracic region of the Alderson phantom. Approximate phantom dimension 30×18cm. The central TLDs were positioned at isocenter.

**Figure 3:**
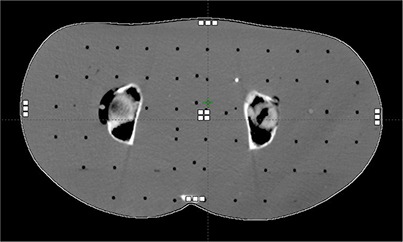
Positions of TLDs (white squares) in the pelvic region of the Alderson phantom. Approximate phantom dimension 37×19cm. The central TLDs were positioned at isocenter.

Harshaw TLD‐100 chips $\left(3\times 3\times 1\;{\text{mm}}^{3}\right)$ are used. Each measurement plane holds a total of 16 TLDs at five positions, as shown in Figs. 1–3. In order not to damage the Alderson phantom, the TLDs are placed inside a 1 cm thick slab of polystyrene, which is positioned between the slices of the phantom. Figures 1–3 show projections of the TLDs to the phantom geometry. Additional nine TLDs served as reference and background dosimeters.

The TL dosimeters are individually calibrated in a C60o beam, at 2 cm depth in a polystyrene phantom. A factor that corrects for the difference between the response of the TLD in the calibration beam quality and the beam quality used for the measurement is applied (Table 3.

**Table 3:** Factor to correct for the difference between the response of the TLD‐100 in the calibration beam quality ${(}^{60}\text{Co})$ and the beam quality used for the measurement.

*Tube Potential [kV]*	*Beam Quality Correction Factor, C*
100	0.74
110	0.76
125	0.78

Average values based on data from Das et al.,^(8)^ Davis et al.,^(9)^ Bankvall G (personal communication), Gonzalez et al.,^(10)^ Nunn et al.,^(11)^ Thilander Klang A (personal communication). Estimated standard uncertainty 7%.

### C. Point doses and dose profiles in a 45 cm long CTDI body phantom

Point doses and dose profiles are measured using the CT Dose Profiler in standard CTDI body phantoms (PMMA cylinder, diameter 32 cm, length 14–15 cm). This type of phantom is designed for measurements in conventional CT scanners with a narrow beam. As the CBCT has a wide beam (13.7 cm, or 15 cm in this work), three CTDI phantoms are joined together, providing a total phantom length of 45 cm, so as not to significantly underestimate the scattered dose for the profiles. Custom made PMMA rods (diameter 12 mm) that run the length of the phantoms are placed in four of the five measurement holes to keep the phantoms in place. One hole at a time is used for measurements.

**Figure 4:**
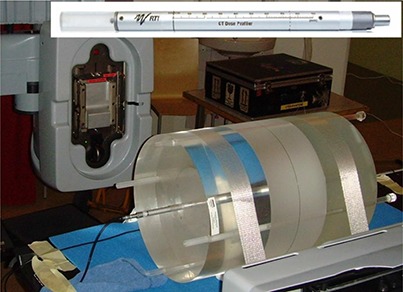
CT Dose Profiler in the measurement setup. Three CTDI body phantoms joined together, with a CT Dose Profiler detector placed in the central hole. Upper right: the CT Dose Profiler.

The CT Dose Profiler is used for measuring the point dose in the central transversal plane of the phantom, as well as dose profiles across the CBCT field. By reducing the measurement time to 1sec, individual pulses from the X‐ray tube are also studied. Figure 4 shows the CT Dose Profiler in the measurement setup. The dose sensitive part of the detector consists of a solid state chip $\left(2\times 2\times 0.25\;{\text{mm}}^{3}\right)$ ^(12)^ that is mounted in a cylindrical tube (165+40mm length, 12.5 mm diameter) that fits in a standard CTDI phantom. The detector is connected with a shielded cable to the electrometer of the RTI readout unit (Barracuda or Piranha) where the charge is collected for each sample (20 bit dynamic range) at a maximum speed of 2000 samples/sec. The software CT Dose Profile Analyzer controls the hardware and collects, calculates, and stores the data from the CT Dose Profiler. It also holds a database that contains correction factors (e.g. phantom, kV) that are automatically applied to the readings. The detector is calibrated fully irradiated in a PTB (Physikalisch‐Technische Bundesanstalt, Germany) traceable field. The calibration factor is 0.3818 mGy/nC for the standard radiation quality RQR 9 (120 kV)^(13)^ for the detector used. The detector response is very fast, linear, and rotation independent,^(^ ^14^ ^,^ ^15^ ^)^ and it can therefore easily track the dynamic variation of the dose rate with both high‐time and spatial resolution. The dose rate waveform can be stored up to a length of 160 sec or down to 320 ms. From the dose rate wave form, the dose is calculated by integrating over a user‐specified time interval. To derive a dose profile, the CT Dose Profiler is moved continuously, with a speed of 10 mm/sec (in this case), by means of a motor that is attached with a thin line. The detector travels the distance through the three phantoms in about 60 seconds, which is about the same time it takes to finish a 360° CBCT scan. To verify the absolute dose measured with the CT Dose Profiler, it is compared to TLD for a few measurement points. Four TLDs are then placed at the same position as the CT Dose Profiler, and a separate scan is made.

### D. Spatial resolution

For both OBI versions, the image quality is evaluated using a Catphan phantom.

## III. RESULTS & DISCUSSION

### A. Dose distribution in an anthropomorphic phantom

Results of the measured dose distribution during a CBCT scan of the Alderson phantom are shown in Tables 4–6. Table 4 shows doses for default imaging modes in OBI v1.3; the average dose is 100 mGy for a Full fan head scan, and 90 mGy for a Half fan pelvis scan. However, local doses are up to 1.4 times higher than the average dose, due to the inhomogeneous dose distribution. A Half fan head scan results in 1.3 times higher average dose than the Full fan head scan.

**Table 4:** Dose distribution in the head and pelvic region of the Alderson phantom, for default CBCT modes of the Varian OBI 1.3 system. Average of three or four TLDs at each position. The CBCT modes in OBI 1.4 that most closely correspond to the CBCT modes in OBI 1.3 are given in parenthesis. CTDI values from the OBI Reference Guide shown for comparison.

*Position*	*CBCT Mode v1.3 Absorbed dose [mGy] (min‐max)*		
	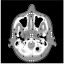 *Standard Dose Full Fan (vl.4: High‐quality head)*	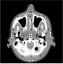 *Standard Dose Half Fan (vl.4 n/a)*	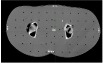 *Standard Dose Half Fan (vl.4: Pelvis)*
Central	**107** *(104‐109)*	**112** *(108‐115)*	**75.2** *(73.6‐76.8)*
Posterior	**84.5** *(82.7‐86.5)*	**119** *(116‐124)*	**96.1** *(94.2‐99.0)*
Lat dx	**101** *(98.9‐104)*	**140** *(138‐141)*	**64.2** *(63.0‐66.0)*
Lat sin	**114** *(111‐115)*	**144** *(139‐147)*	**74.0** *(73.1‐75.7)*
Anterior	**84.9** *(83.9‐85.9)*	**139** *(136‐141)*	**125** *(124‐126)*
**Average [mGy]**	**98**	**130**	**87**
${\text{CTDI}}_{W}\;[\text{mGy}]$ ^a^	**90**	*n/a*	**38**

^a^ Data from the Varian On‐Board Imager (OBI) Reference Guide.

**Table 6:** Dose distribution in the thoracic and pelvic region, respectively, of the Alderson phantom, for default CBCT modes of the Varian OBI 1.4 system. Average of three or four TLDs at each position. CTDI values from the OBI Reference Guide shown for comparison.

***Thorax / Pelvis***	*CBCT Mode v1.4 Absorbed Dose [mGy] (min‐max)*		
*Position*	*Low‐dose Thorax*	*Pelvis Spot Light*	*Pelvis*
Central	**11.7** *(11.4‐11.9)*	**22.4** *(21.1‐23.8)*	**31.5** *(30.5‐31.8)*
Posterior	**10.9** *(10.7‐11.0)*	**50.9** *(59.5‐52.3)*	**38.6** *(38.3‐38.9)*
Lat dx	**8.9** *(8.8‐9.0)*	**31.6** *(31.4‐32.0)*	**25.2** *(24.6‐25.9)*
Lat sin	**10.1** *(9.9‐10.2)*	**17.8** *(16.6‐19.2)*	**28.5** *(28.2‐29.0)*
Anterior	**16.4** *(15.9‐16.6)*	**5.2** *(5.0‐5.4)*	**47.1** *(46.6‐47.6)*
**Average [mGy]**	**11.6**	**25.6**	**34.2**
${\text{CTDI}}_{W}\;[\text{mGy}]$ ^a^	**4.7**	**14.4**	**17.7**

^a^ Data from the Varian On‐Board Imager (OBI) Reference Guide.

The combined uncertainty of the TLD measurements is estimated to about ±10%, including the uncertainty of the beam quality factor, and statistical measurement uncertainty. The measured data typically agrees with previously published data. Li et al.^(1)^ measured skin doses up to 100 mGy. Song et al.^(3)^ reported an average dose of 83 mGy for a Full fan head scan. The highest dose measured by Wen et al.^(2)^ was to the hip joint, at 110 mGy. Imaging doses in this range are likely to limit the number of CBCTs that can be performed during the patient's course of treatment.

Tables 5 and 6 show the results of the measurements for default imaging modes in OBI v1.4. It is clear that the doses in OBI v1.4 are significantly lower than in v1.3; point doses measured in the latter range between 64 mGy and 144 mGy, compared to between 1 mGy and 51 mGy in v1.4. This is also evident when comparing the CBCT modes in OBI 1.4 that most closely correspond to the CBCT modes in OBI 1.3 (see Table 4). The average dose in the Full fan head scan is reduced by a factor of 4 in OBI v1.4 compared to the earlier version, according to the measurements. For a Half fan pelvis scan, the dose is 2.5 times lower. The dose reduction is a result of a reduction in the exposure parameters and the introduction of the 200° scan option.

**Table 5:** Dose distribution in the head region of the Alderson phantom, for default CBCT modes of the Varian OBI 1.4 system. Average of three or four TLDs at each position. CTDI values from the OBI Reference Guide shown for comparison.

***Head***	*CBCT Mode vl.4 Absorbed Dose [mGy] (min‐max)*		
*Position*	*Low Dose Head*	*Standard Dose Head*	*High‐quality Head*
Central	**2.7** *(2.6‐2.8)*	**4.9** *(4.65‐5.12)*	**24.7** *(23.8‐25.1)*
Posterior	**3.3** *(3.2‐3.3)*	**6.3** *(6.20‐6.34)*	**30.7** *(30.6‐30.8)*
Lat dx	**2.5** *(2.5‐2.5)*	**6.3** *(6.17‐6.36)*	**30.7** *(30.1‐31.1)*
Lat sin	**3.4** *(3.3‐3.4)*	5.1 *(4.96‐5.26)*	**25.5** *(24.6‐26.0)*
Anterior	**0.8** *(0.8‐0.8)*	**1.5** *(1.46‐1.56)*	**7.9** *(7.7‐8.1)*
**Average [mGy]**	**2.5**	**4.8**	**23.9**
${\text{CTDI}}_{W}\;[\text{mGy}]$ ^a^	**2.0**	**3.9**	**19.4**

^a^ Data from the Varian On‐Board Imager (OBI) Reference Guide.

According to the TLD measurements, the three head scan modes in OBI v1.4 result in average doses of 2.5 mGy, 5 mGy, and 25 mGy per scan, respectively (i.e. a factor of 10 difference). Which mode to use naturally depends on the required image quality. The Low‐dose thorax mode gives an average dose of 12 mGy per scan, and a CBCT scan of the pelvis, 25 mGy to 35 mGy. Local doses are up to 2 times higher, due to the inhomogeneous dose distribution.

Comparing the five measurement points, the dose in the head region is quite uniform, with a max/min ratio of 1.3, irrespective of OBI version (if the anterior point of v1.4 is exempted, see below). As the phantom size increases, the dose is more heterogeneous; the max/min ratio is close to 2 for thorax and pelvis, for both OBI versions, and 3 for the pelvis spot light (if the anterior point is exempted). For pelvis, the dose is higher at the anterior/posterior positions than at the lateral positions, due to the combined effects of incident and transmitted/scattered radiation.

For the scan modes that use a 360° acquisition angle, the dose at the left side of the phantom is slightly higher than at the right side. The X‐ray beam starts and ends at the left side, and the effect is caused by a small overscan.^(16)^

Tables 5 and 6 (3rd column) clearly show the dose reduction in the anterior part due to the 200° scan, which avoids imaging at angles $\pm 80^{{}^{\circ}}$ from the front. In the head, the anterior measurement point receives about one‐quarter of the maximum dose measured in the plane, which is favorable for the lens which has a relatively low tolerance dose of about 12 Gy for fractionated treatments.^(17)^ For pelvis spot light, the anterior dose is one‐tenth of the maximum.

CTDI is sometimes considered an estimate of the patient dose. In the head region, the measured average dose agrees within the uncertainty with ${\text{CTDI}}_{w}$ data reported by Varian, for both OBI versions. However, for thorax and pelvis, the measured average dose is about twice as high as the data reported by Varian. ${\text{CTDI}}_{w}$ is defined for either a 16 cm (‘head’) or a 32 cm (‘body’) diameter cylindrical PMMA phantom, 15 cm in length. The transverse dimensions of the head region of the Alderson phantom are similar to the CTDI head phantom, while the dimensions of the thoracic and pelvic region differ somewhat from the CTDI body phantom. Differences in phantom geometry and composition likely contribute to the dose differences. Also, the average doses as presented in Tables 4–6 are averages of the five measurement points in the plane, while the ${\text{CTDI}}_{w}$ concept weights the peripheral points by two‐thirds and the central point by one‐third.

### B. Point doses and dose profiles in a 45 cm long CTDI body phantom

Point doses due to CBCT scans of a 45 cm long CTDI phantom, measured using TLD, are shown in Table 7. Except for the phantom length, the phantom geometries in this case are similar, when comparing the measured mean dose with ${\text{CTDI}}_{w}$ data reported by Varian. As expected, the dose differences are smaller than for the anthropomorphic phantom, but still significant; weighted average dose (1/3central+2/3peripheral)=29.2mGy, ${\text{CTDI}}_{w}=17.7\;\text{mGy}$.

**Table 7:** Measured point dose in a 45 cm long CTDI body phantom, for default CBCT modes of the Varian OBI 1.4 system. Weighted average dose (1/3central+2/3peripheral) is calculated based on assuming a constant peripheral dose.

***Detector***	***Thorax/Pelvis** Position*	*Low‐dose Thorax*	*CBCT Mode v1.4 Absorbed Dose [mGy] Pelvis Spot Light*	*Pelvis*
**TLD**	Central	**5.6**	**19.2**	**21.4**
	Peripheral (12 o'clock)	*n/a*	*n/a*	**33.1**
	**Weighted average [mGy]**	*n/a*	*n/a*	**29.2**
**CT Profiler**	Central	**5.3**	**16.6**	**19.0**
	Peripheral (12 o'clock)	*n/a*	*n/a*	**28.2**
	**Weighted Average [mGy]**	*n/a*	*n/a*	**25.1**
	${\text{CTDI}}_{W}\;[\text{mGy}]$ ^a^	**4.7**	**14.4**	**17.7**

^a^ CTDI values from the OBI Reference Guide shown for comparison.

Table 7 also shows point doses measured with the CT Profiler. The CT Profiler data is approximately 5–10% lower than the TLD measurements, which is within the uncertainty of the measurements.

**Figure 5:**
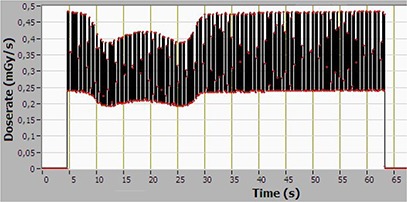
Time‐varying point dose rate at the center of a 45 cm long CTDI body phantom, recorded with the CT Profiler, for the pelvis scan mode.

Figure 5 shows the time‐varying average point dose rate at the center of a 45 cm long CTDI phantom, recorded with the CT Profiler, for the pelvis scan mode. Integration over the entire scan time gives the absorbed dose. The reduced signal for the 10–30 sec time period is due to attenuation in the treatment table (iBEAM, Elekta).

**Figure 6:**
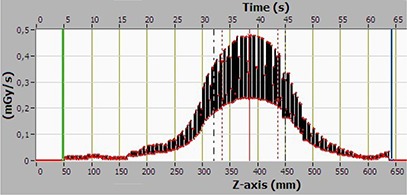
Dose rate profile along the central axis of a 45 cm long CTDI body phantom, recorded with the CT Profiler, for the pelvis scan mode.

Dose profile along the central axis for the same case is shown in Fig. 6. A small reduction in the signal can be observed at T approx/equal to 170 sec, as the CT Profiler probe exits the phantom, and continues to measure in air. It is clear that three phantoms are required so as not to significantly underestimate the scattered dose when measuring dose profiles for a beam width of 15 cm.

**Figure 7:**
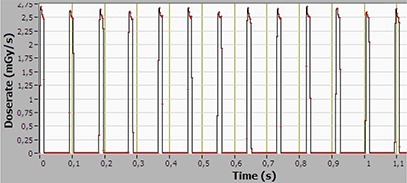
Instantaneous dose rate at the center of a 45 cm long CTDI phantom, recorded with the CT Profiler, for the pelvis scan mode.

**Figure 8:**
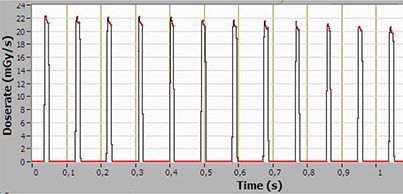
Instantaneous dose rate at the periphery of the midplane of a 45 cm long CTDI phantom, recorded with the CT Profiler, for the pelvis scan mode.

Unlike conventional CT, the X‐ray beam in the OBI system is pulsed with a frequency of about 10 Hz (synchronized with the readout of the flat‐panel imager). The pulses are seen in Fig. 5, which displays the point average dose rate. The individual X‐ray pulses cannot be resolved fully due to the long measurement time. To resolve the instantaneous dose rate, the measurement time is reduced to 1 sec in Figs. 7 and 8. It is interesting to note that the dose rate at the periphery of the phantom (1 cm depth) is 22 mGy/sec; approximately 10 times higher than at the center. Dose rates at this level have been shown to influence the operation of implantable cardiac rhythm management devices (ICRMD) during CT irradiation.^(18)^ The reported effects were predominantly transient, and associated only with direct irradiation. In a CT scanner, the dwell time of the radiation over the electronics module of the ICRMD is in the order of seconds, compared to 1 min for a 360° CBCT scan. However, as it is well known that radiotherapy may cause ICRMD to malfunction due to the effects of ionizing radiation or electromagnetic interference,^(19)^ it is quite unlikely that ICRMD will be present in the volume irradiated during CBCT.

### C. Spatial resolution

The measured spatial resolution in v1.3 is 10 lp/cm and 8 lp/cm for standard dose Full fan and standard dose Half fan, respectively, which is better than the specification (7 lp/cm and 6 lp/cm, respectively). The spatial resolution in v1.4 is not as high; 8 lp/cm and 5 lp/cm for the corresponding CBCT modes. Also the specification has been reduced, from 6 lp/cm in v1.3 to 4 lp/cm in v1.4 for the standard dose Half fan / pelvis mode.

## IV. CONCLUSIONS

Measurements show that the imaging doses for default modes in Varian OBI v1.4 CBCT system are significantly lower than in v1.3. In v1.3 the mean dose is around 100 mGy (point dose maximum 150 mGy). In v1.4 mean doses range between 3–35 mGy (point dose maximum 50 mGy), depending on CBCT mode. Instantaneous dose rate can be higher than 20 mGy/sec. Compared to v1.3, the spatial resolution in v1.4 has been compromised to lower the dose. The CT Dose Profiler is proven fast and accurate for CBCT applications. Three CTDI phantoms are required when measuring the dose profile, so as not to significantly underestimate the scattered dose from these wide beams.

## ACKNOWLEDGEMENTS

MTF/Diagnostik at Sahlgrenska University Hospital, Göteborg, and BFD Engineering and physics at Södra Älvsborg Hospital, Borås, are acknowledged for providing CTDI phantoms.
